# Supplementary material for: Vitrification-enabled enhancement of proton conductivity in hydrogen-bonded organic frameworks
Source: Nat Commun. 2024 May 10;15:3930. doi: 10.1038/s41467-024-48158-8 (PMC11087529; doi:10.1038/s41467-024-48158-8)
Supplement: Supplementary file 1 — Supplementary Information [file 41467_2024_48158_MOESM1_ESM.pdf]

Supplementary Information

Vitrification-Enabled Enhancement of Proton Conductivity in Hydrogen-Bonded Organic Frameworks

Feng-Fan Yang,[1] Xiao-Lu Wang,[1, 2] Jiayue Tian,[3] Yang Yin,[1] Linfeng Liang[1*]
[1]*Institute of Crystalline Materials, Shanxi University, Taiyuan 030006, Shanxi, China.* [2]*College of Chemistry, Taiyuan University of Technology, Taiyuan, 030024, China.* [3]*School of Materials and mhemical Engineering, Zhengzhou University of Light Industry, Zhengzhou,450001, China.* Correspondence and requests for materials should be addressed to Linfeng Liang (email: jtcl@sxu.edu.cn)

Contents

Section 1. Supplementary Tables..S2

Section 2. Supplementary Figures..S8

Section 3 Supplementary References..................................S23

Section 1 Supplementary Tables

Supplementary Tab. 1 Crystallographic parameters of **HOF-SXU-8**.

Compound	**HOF-SXU-8**
CCDC	2292290
Empirical formula	$C_{16}H_{26}N_2O_{10}S_2$
Formula weight	470.51
Temperature (K)	293(2)
Crystal system	monoclinic
Space group	$P2_1/n$
a (Å)	9.1601(3)
b (Å)	12.0981(4)
c (Å)	10.0596(4)
α (°)	90
β (°)	100.113(10)
γ (°)	90
Volume(Å^3)	1097.48(7)
Z	2
D_c (g cm^{-3})	1.424
μ (mm^{-1})	0.297
F (000)	496.0
Radiation	Mo-Kα (λ = 0.71076)
Goodness-of-fit on F^2	1.036
Final R indexes [I>=2σ (I)][a]	R_1 = 0.0412, wR_2 = 0.1146
Final R indexes [all data] [a]	R_1 = 0.0466, wR_2 = 0.1186
Largest diff. peak/hole / e Å^{-3}	0.55/-0.32

$R_1 = \sum||F_0| - |F_c||/\sum|F_0|;\ wR_2 = [\sum w\,(|F_0|^2 - |F_c|^2)^2/\sum w\,({F_0}^2)^2]^{1/2}$

Supplementary Tab. 2 Crystallographic parameters of **HOF-SXU-9**.

Compound	**HOF-SXU-9**
CCDC	2292291
Empirical formula	$C_{14}H_{22}N_2O_6S_2$
Formula weight	378.46
Temperature (K)	296.00
Crystal system	monoclinic
Space group	$P2_1/c$
a (Å)	11.8976(8)
b (Å)	9.6259(7)
c (Å)	16.1176(12)
α (°)	90
β (°)	94.218(2)
γ (°)	90
Volume(Å^3)	1840.9(2)
Z	4
D_c (g cm^{-3})	1.366
μ (mm^{-1})	0.320
F (000)	800.0
Radiation	Mo-Kα (λ = 0.71073)
Goodness-of-fit on F^2	1.059
Final R indexes [*I*>=*2σ (I)*][a]	R_1 = 0.0384, wR_2 = 0.1056
Final R indexes [all data] [a]	R_1 = 0.0453, wR_2 = 0.1114
Largest diff. peak/hole / e Å^{-3}	0.32/-0.35

$R_1 = \sum||F_0| - |F_c||/\sum|F_0|$; $wR_2 = [\sum w\,(|F_0|^2 - |F_c|^2)^2/\sum w\,({F_0}^2)^2]^{1/2}$

Supplementary Tab. 3 Structure and distances of hydrogen bonds in **HOF-SXU-8**.

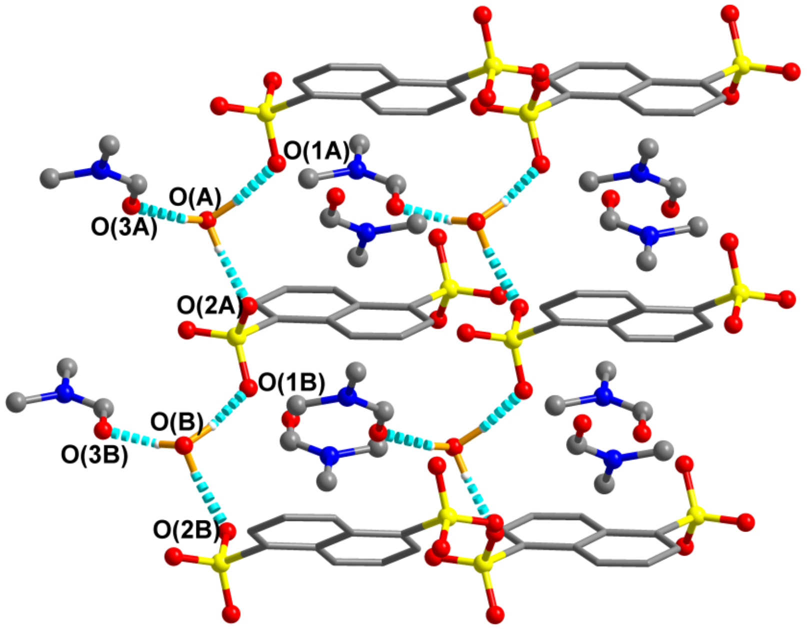

Hydrogen Bonds	Distances (Å)	Angles (°)
O(A)···O(1A)	2.65 Å	166.85
O(A)···O(2A)	2.65 Å	152.37
O(A)···O(3A)	2.44 Å	161.86
O(B)···O(1B)	2.65 Å	166.85
O(B)···O(2B)	2.65 Å	152.37
O(B)···O(3B)	2.44 Å	161.86

Supplementary Tab. 4 Structure and distances of hydrogen bonds in **HOF-SXU-9**.

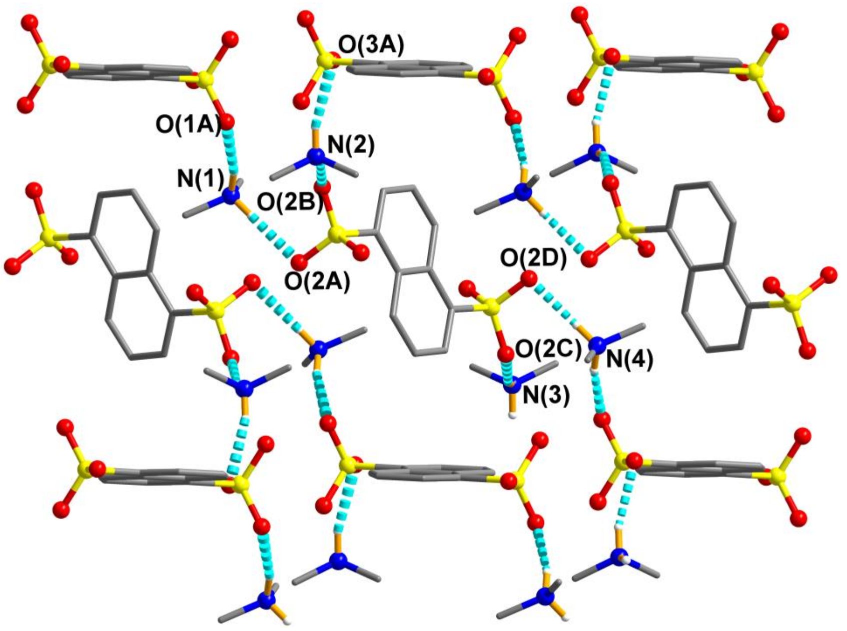

Hydrogen Bonds	Distances (Å)	Angles (°)
N(1)···O(1A)	2.80 Å	166.62
N(1)···O(2A)	2.88 Å	177.00
N(2)···O(3A)	2.93 Å	164.13
N(2)···O(2B)	2.75 Å	158.75
N(3)···O(2C)	2.75 Å	158.75
N(4)···O(2D)	2.88 Å	177.00

Supplementary Tab. 5 Elemental analysis.

	C	H	N	S
**HOF-SXU-8**				
Calculated:	40.74%	5.68%	6.15%	13.31%
Found:	40.70%	5.48%	6.17%	13.51%
**HOF-g**				
Calculated:	39.94%	5.29%	4.90%	14.59%
Found:	39.14%	5.21%	4.94%	14.63%
**HOF-SXU-9**				
Calculated:	44.43%	5.86%	7.40%	26.94%
Found:	44.29%	5.43%	7.55%	16.72%

Supplementary Tab. 6 Summary of the state-of proton conductors based on HOFs.

	σ (S cm^{-1})	Conditions	Ea (eV)	Ref.
HOF-6a	1.9×10^{-6}	40°C (97%RH)	-	1
HOF-GS-10	7.5×10^{-3}	30°C (95%RH)	0.489	2
HOF-GS-11	1.8×10^{-2}	30°C (95%RH)	0.135	2
CPOS-1	1.0×10^{-2}	60°C (98%RH)	0.93	3
CPOS-2	2.2×10^{-2}	60°C (98%RH)	0.61	3
CPOS-3	3.3×10^{-4}	60°C (98%RH)	0.62	3
CPOS-4	7.4×10^{-4}	60°C (98%RH)	0.82	3
HOF-H_3L	6.91×10^{-5}	100°C (98%RH)	0.68	4
UPC-H1	5.0×10^{-3}	80°C (95%RH)	0.42	5
UPC-H2	2.6×10^{-3}	80°C (95%RH)	0.79	5
UPC-H3	4.3×10^{-2}	80°C (95%RH)	0.39	5
UPC-H5	1.71×10^{-3}	80°C (95%RH)	0.23	6
HOF 1	3.11×10^{-4}	70°C (98%RH)	0.71	7
HOF 2	4.32×10^{-4}	50°C (98%RH)	0.39	7
HDSD-1	7.50×10^{-3}	80°C (80%RH)	0.44	8
BPPA	5.14×10^{-2}	80°C (95%RH)	0.17	9
HOL-DMSO	4.42×10^{-2}	120°C (without humidity)	0.42	10
HOF-SXU-1	6.32×10^{-3}	160°C (without humidity)	0.16	11
HOF-g	5.62×10^{-2}	100°C (without humidity)	0.23	This work

Section 2 Supplementary Figures

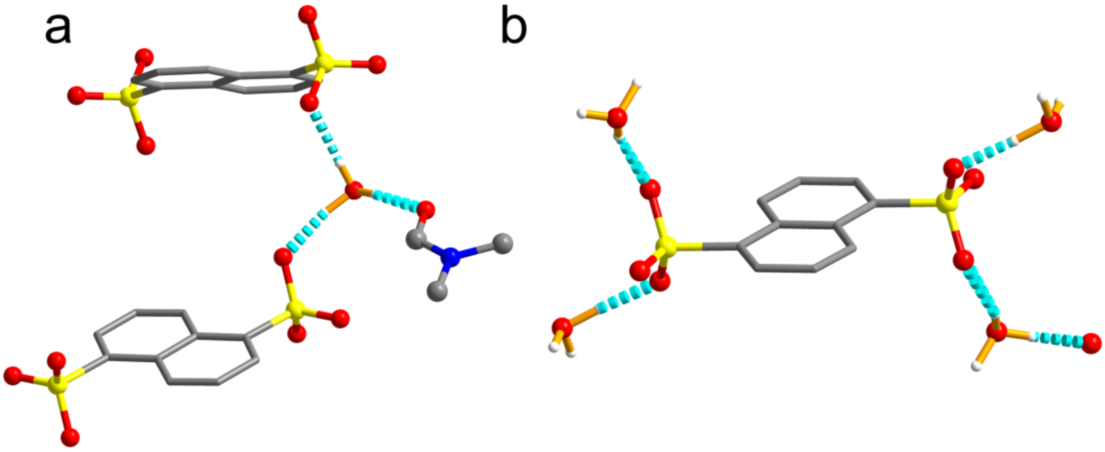

Supplementary Fig. 1. a Hydrogen bonding of H_3O^+ in **HOF-SXU-8**. **b** Hydrogen bonding of 1,5-NSA anion in **HOF-SXU-8**.

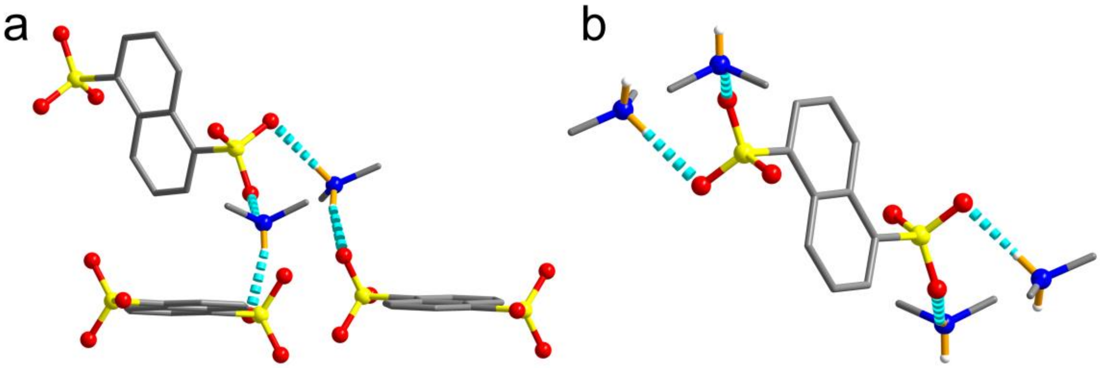

Supplementary Fig. 2. a Hydrogen bonding of Dimethylamine cation in **HOF-SXU-9**. **b** Hydrogen bonding of 1,5-NSA anion in **HOF-SXU-9**.

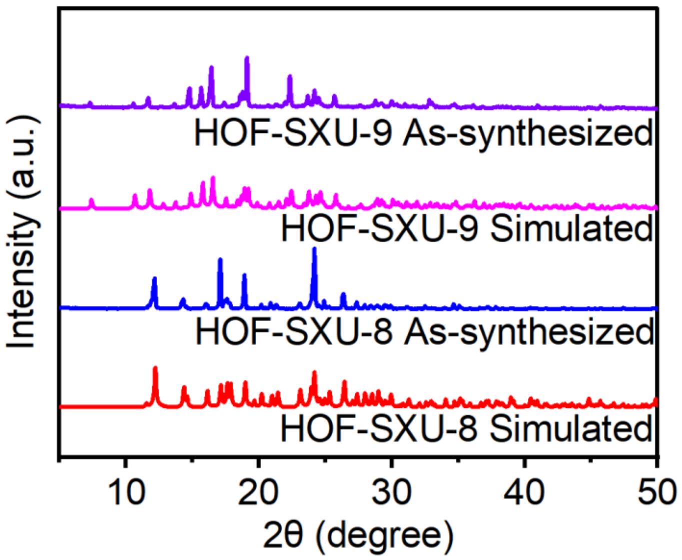

Supplementary Fig. 3. Simulated and as-synthesized powder X-ray diffraction pattern of **HOF-SXU-8** and **HOF-SXU-9**.

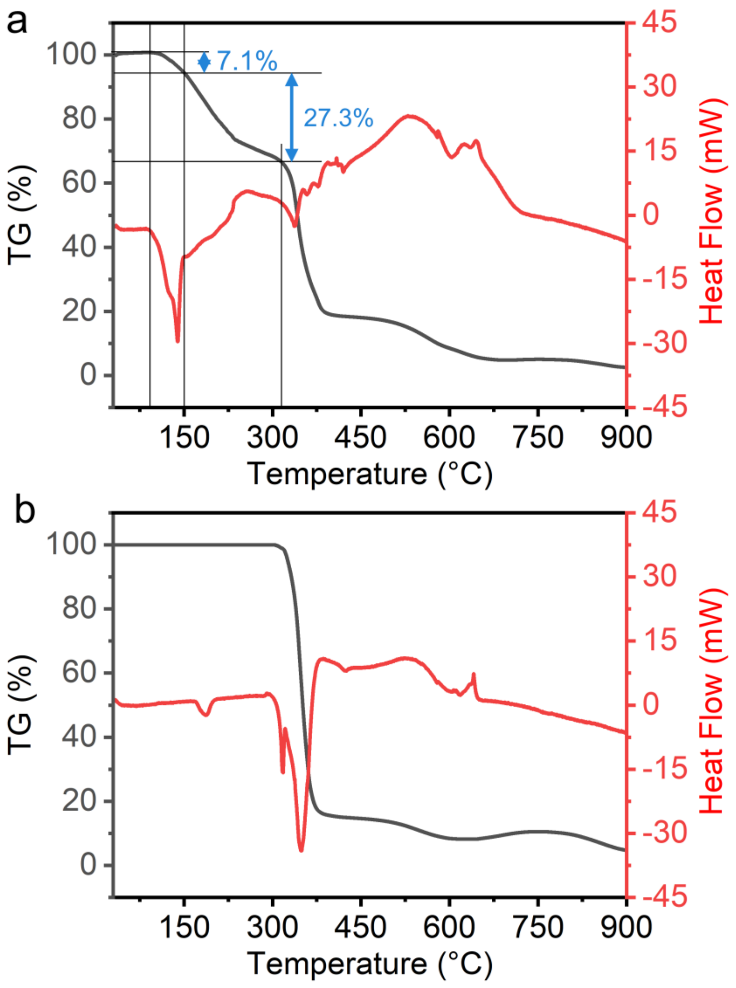

Supplementary Fig. 4. a TG and heat flow of **HOF-SXU-8**. **b** TG and heat flow of **HOF-SXU-9**.

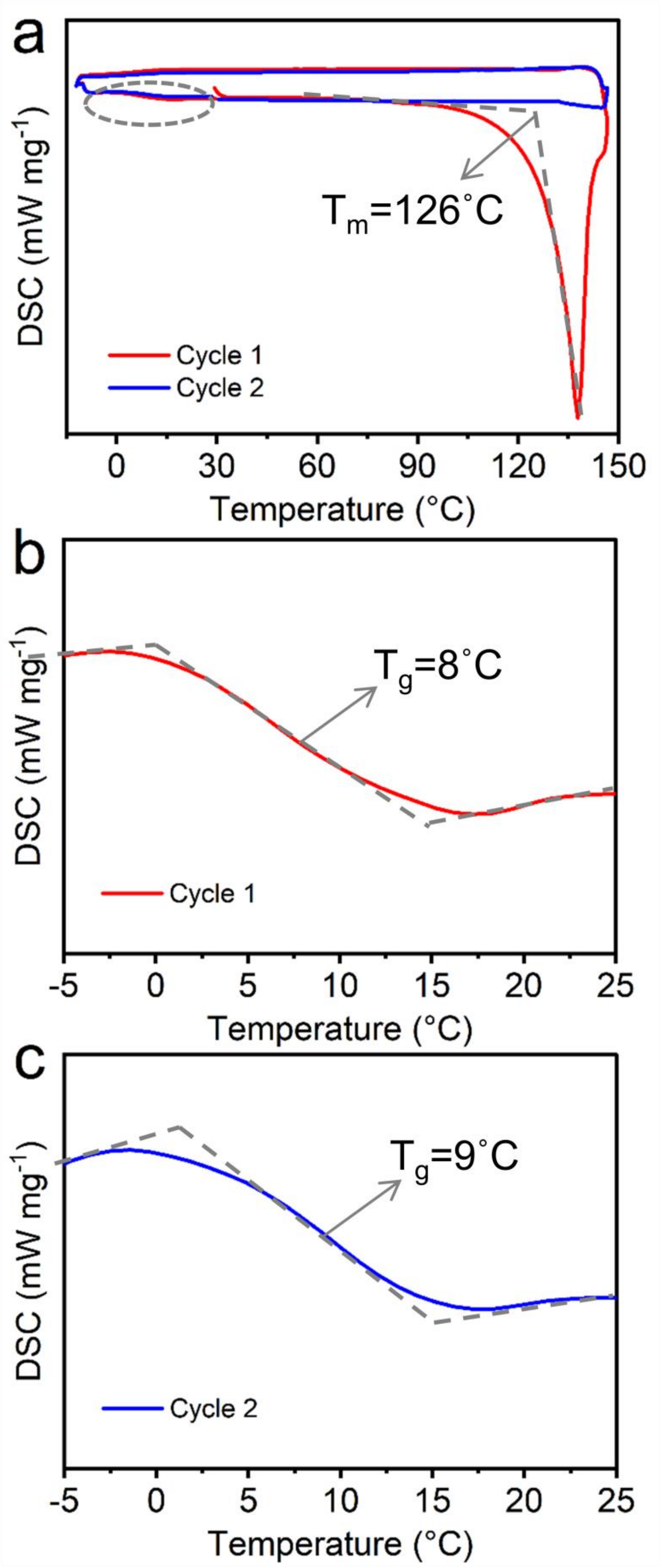

Supplementary Fig. 5. a DSC scans of crystalline **HOF-SXU-8** from -10 to 145°C (red curves represent the first heating and cooling cycles, blue curves represent the second cycle). **b-c** Enlarged figures of the circled part in Supplementary Fig. 5a.

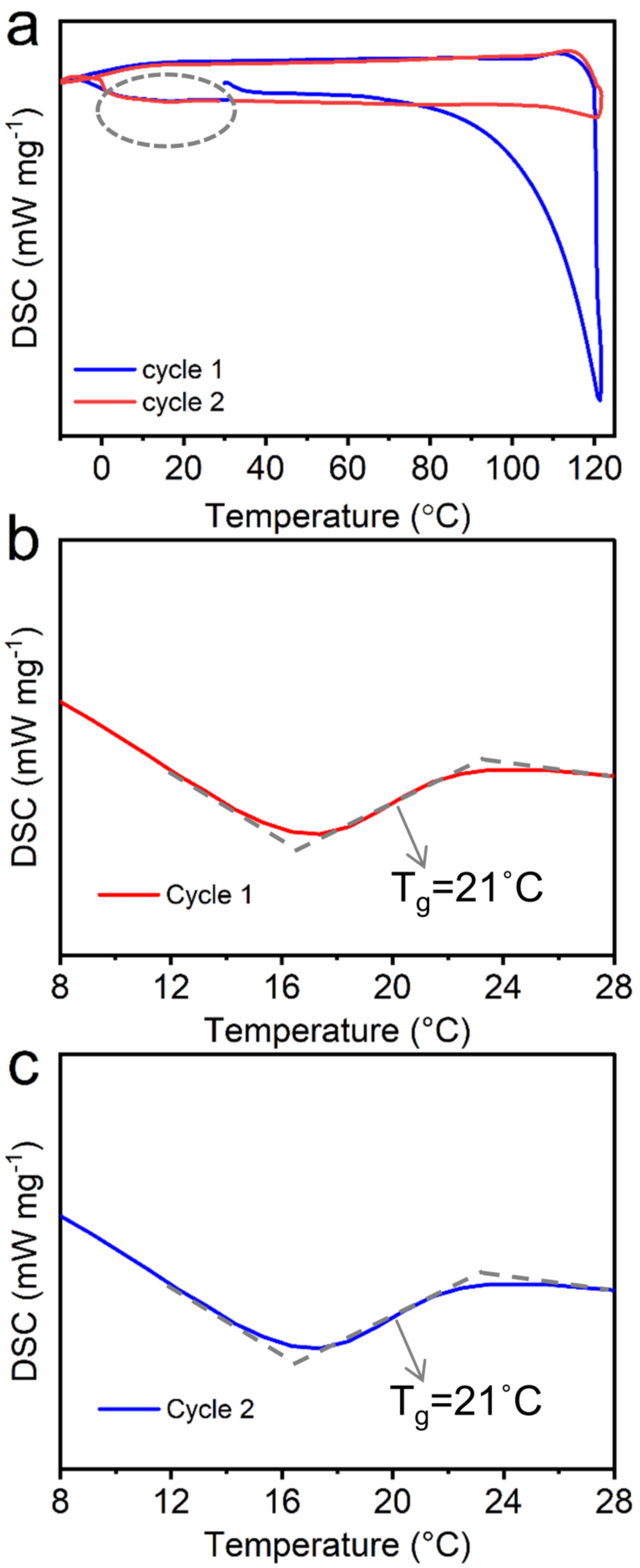

Supplementary Fig. 6. a DSC scans of crystalline **HOF-SXU-8** from -10 to 110°C (red curves represent the first heating and cooling cycle, blue curves represent the second cycle). **b-c** Enlarged figures of the circled part in Supplementary Fig. 6a.

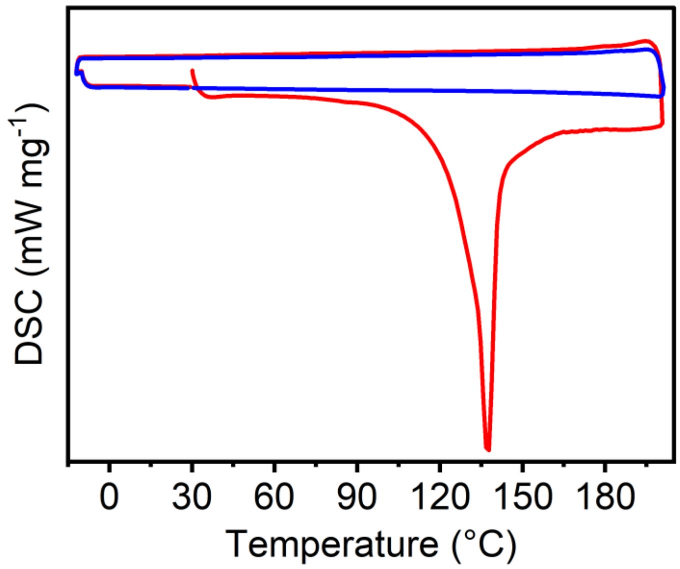

Supplementary Fig. 7. DSC scans of crystalline **HOF-SXU-8** from -10 to 200°C (red curves represent the first heating and cooling cycle, blue curves represent the second cycle).

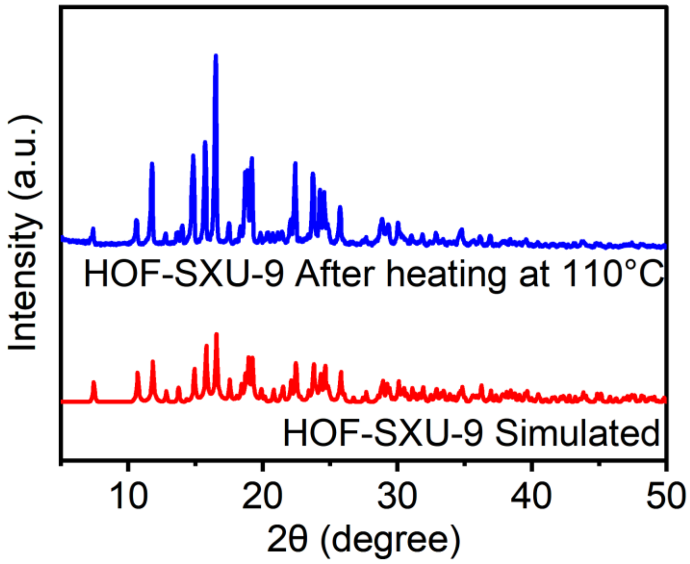

Supplementary Fig. 8. Powder X-ray diffraction pattern of **HOF-SXU-9** after heating at 110°C.

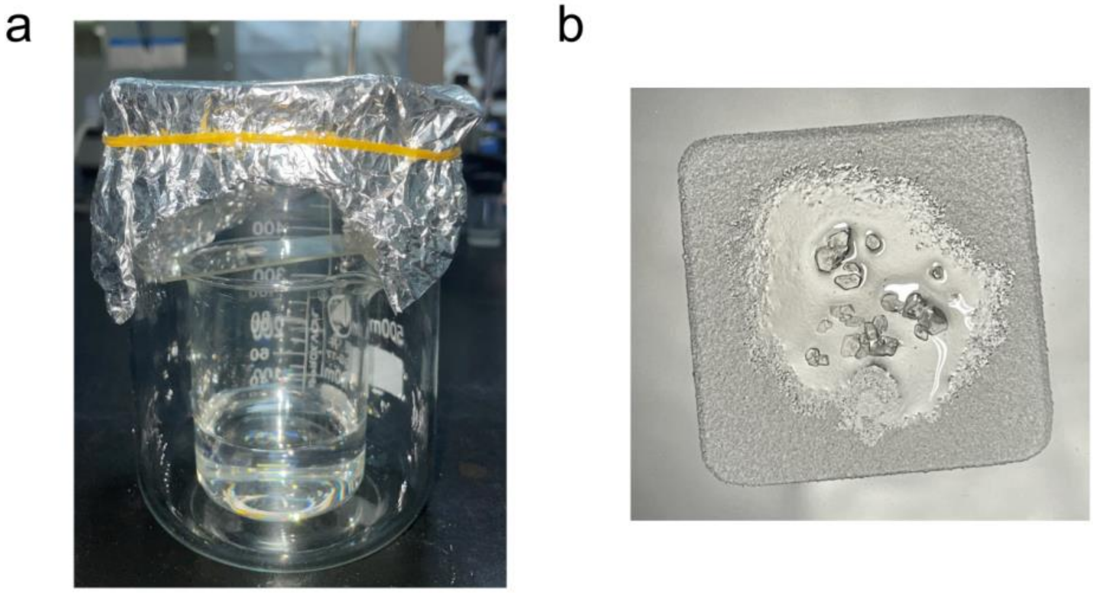

Supplementary Fig. 9. a Diagram of **HOF-g** immersed in the DMF vapor atmosphere. **b** Photograph of **HOF-g** recrystallized after heating and cooling at 110°C in DMF atmosphere.

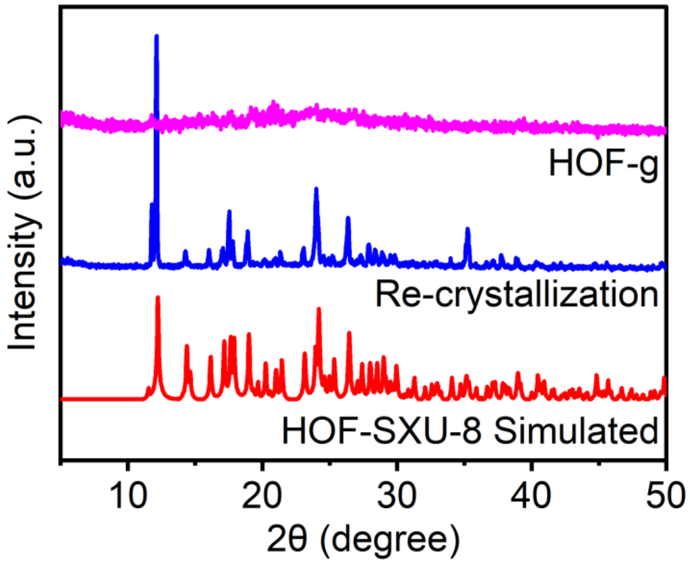

Supplementary Fig. 10. Powder X-ray diffraction pattern of **HOF-g** before (purple) and after (blue) heating at 110°C and cooling in organic solvent DMF.

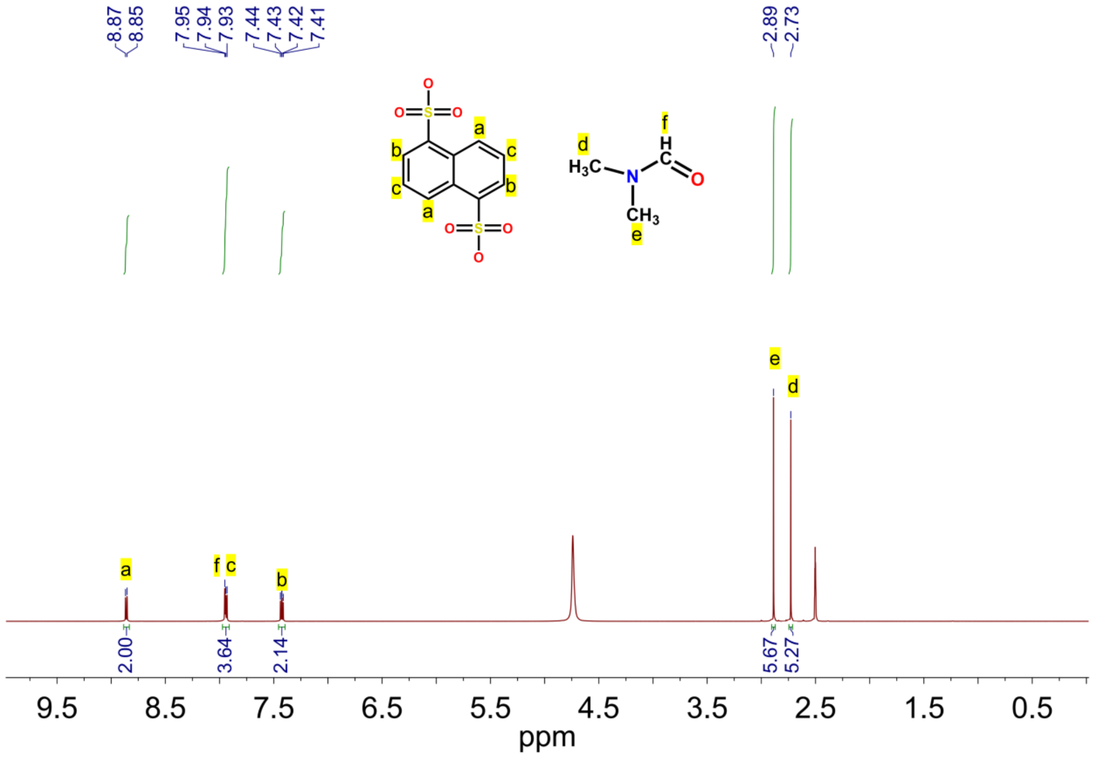

Supplementary Fig. 11. ^{1}H NMR of **HOF-SXU-8** in DMSO-d_6 at room temperature.

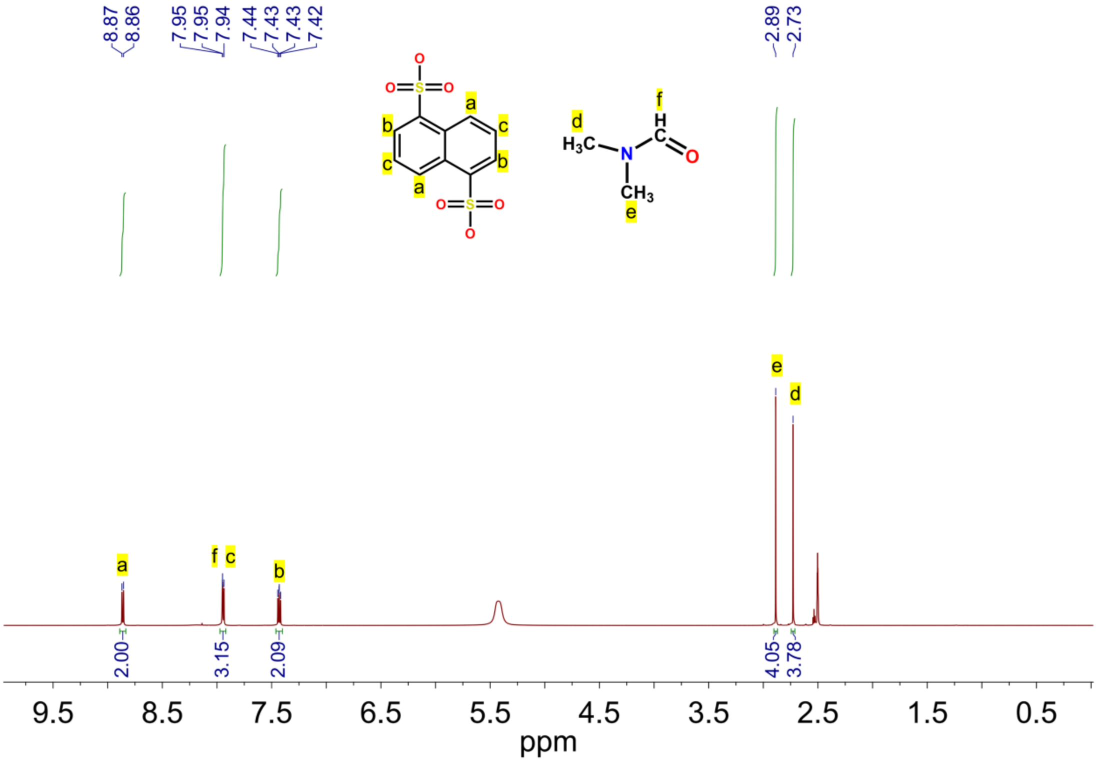

Supplementary Fig. 12. ^{1}H NMR of **HOF-g** in DMSO-d_6 at room temperature.

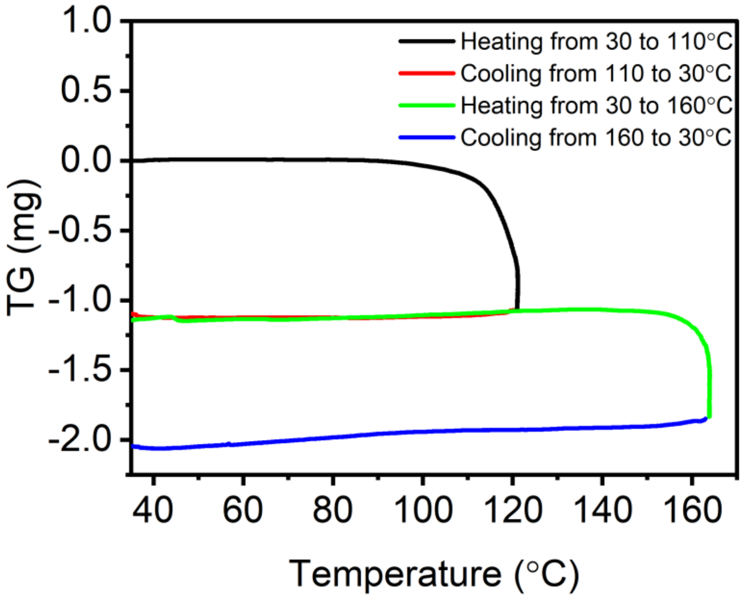

Supplementary Fig. 13. The two rounds heating and cooling cycles TG curve of **HOF-SXU-8**.

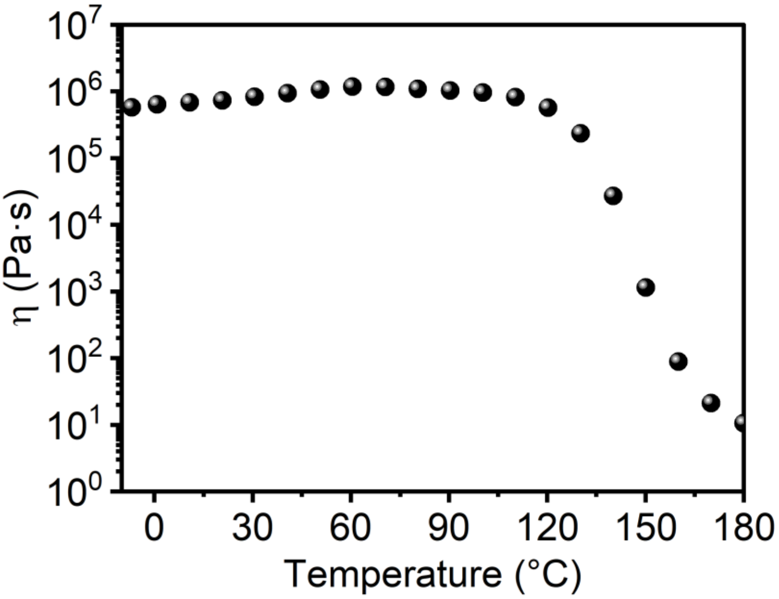

Supplementary Fig. 14. Temperature-dependent viscosity of **HOF-g**.

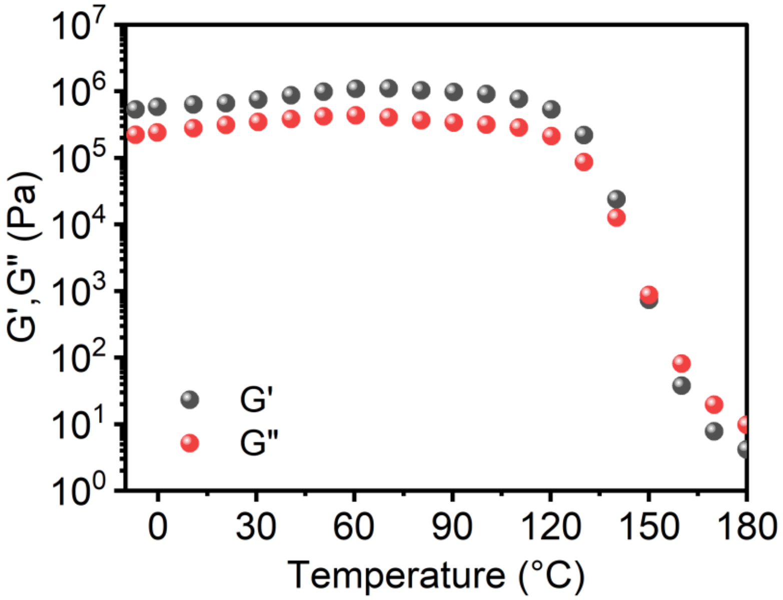

Supplementary Fig. 15. DMA of **HOF-g** from -10 to 180°C (heating rate of 5°C min^{-1}). The storage (G') and loss (G") moduli were marked as black and red, respectively.

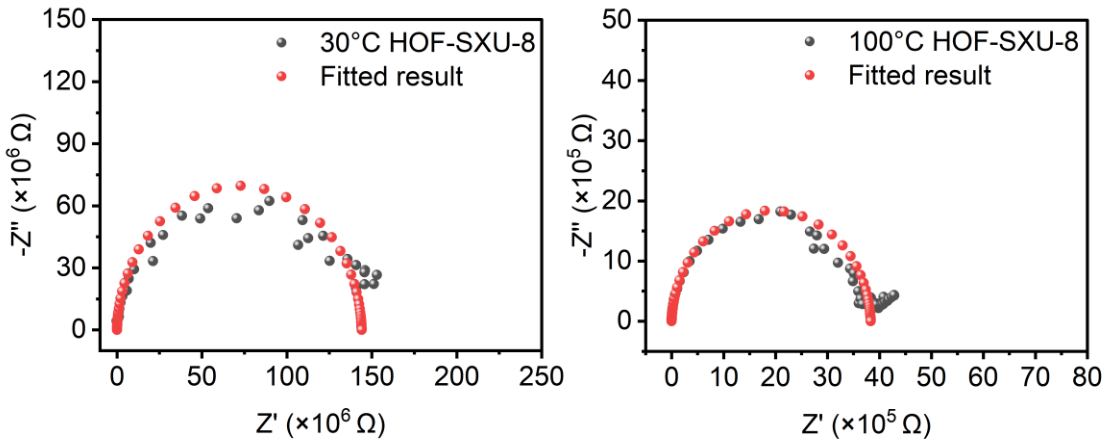

Supplementary Fig. 16. Nyquist plots and fitted results of **HOF-SXU-8** at 30 and 100°C.

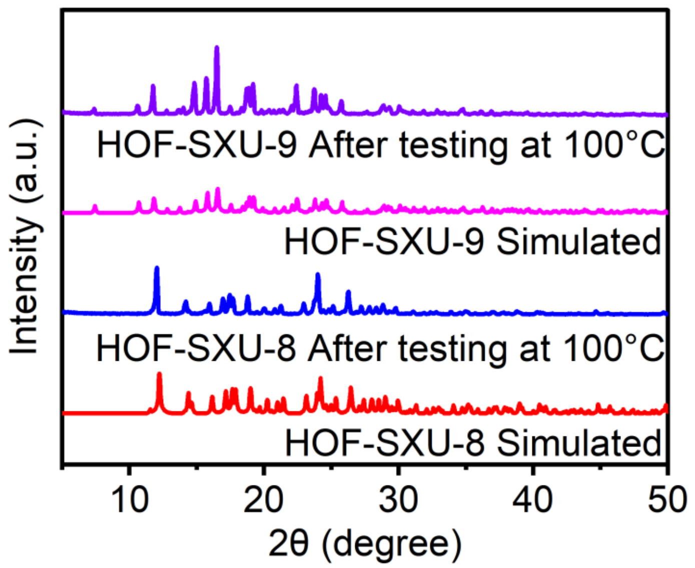

Supplementary Fig. 17. Powder X-ray diffraction patterns of **HOF-SXU-8** and **HOF-SXU-9** after proton conduction measurement at 100°C.

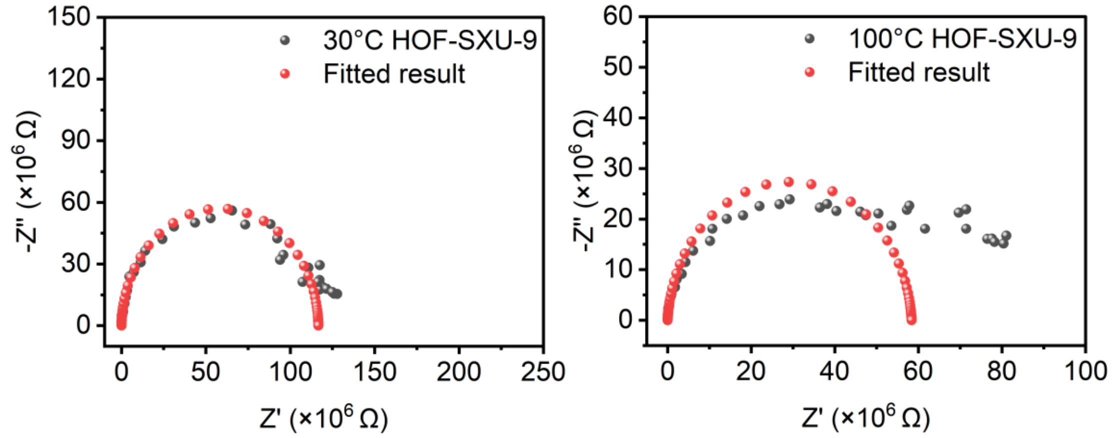

Supplementary Fig. 18. Nyquist plots and fitted results of **HOF-SXU-9** at 30 and 100°C.

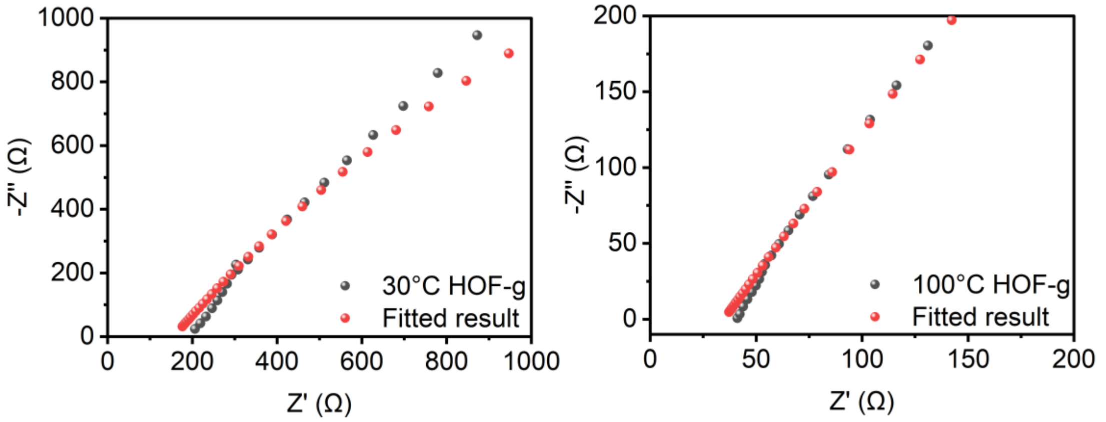

Supplementary Fig. 19. Nyquist plots and fitted results of **HOF-g** at 30 and 100°C.

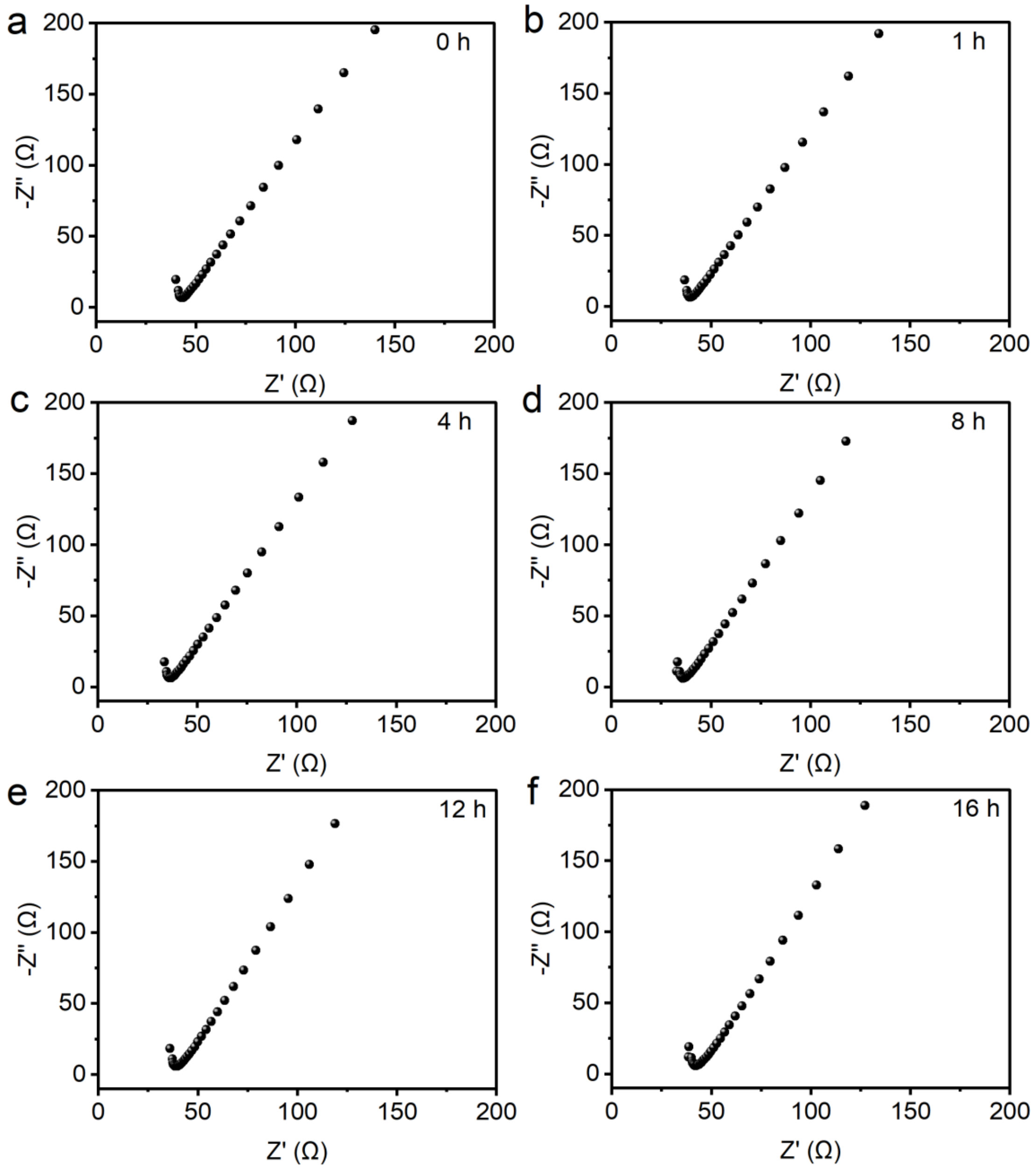

Supplementary Fig. 20. Nyquist plots of **HOF-g** of after **a** 0 h, **b** 1 h, **c** 4 h, **d** 8 h, **e** 12 h, **f** 16 h continuous run at 100°C.

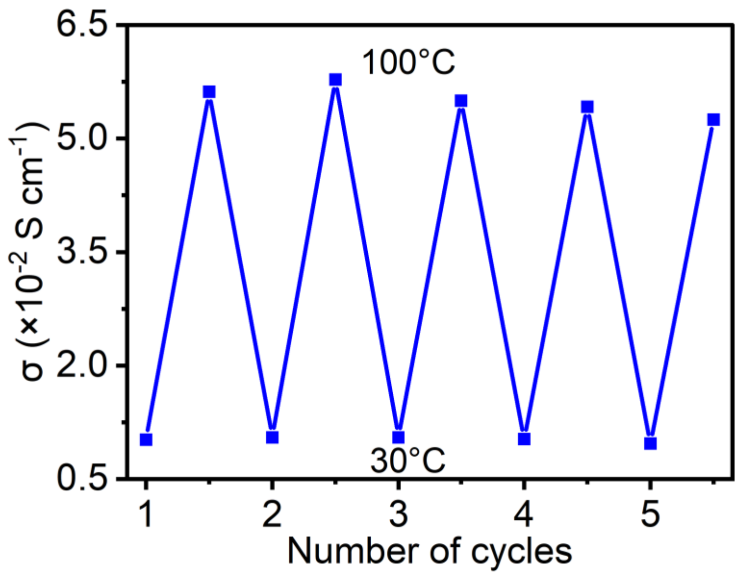

Supplementary Fig. 21. Proton conductivities of **HOF-g** measured across five heating and cooling cycles.

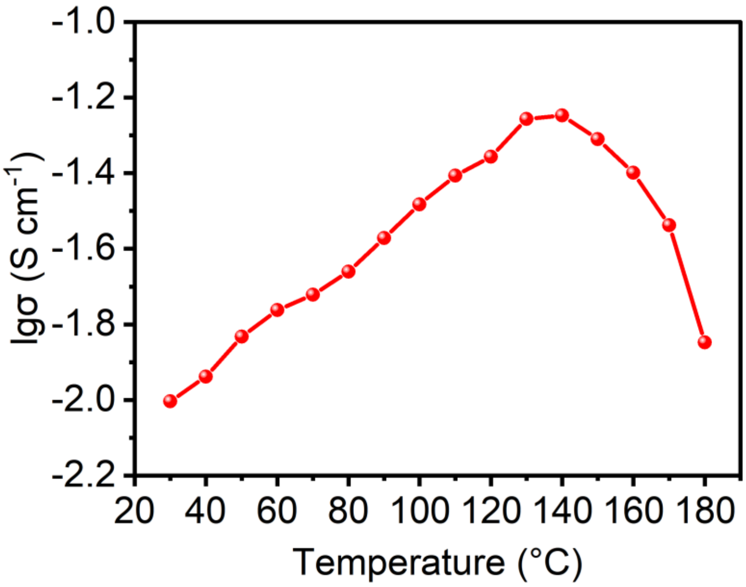

Supplementary Fig. 22. Proton conductivities of **HOF-g** with temperature increasing.

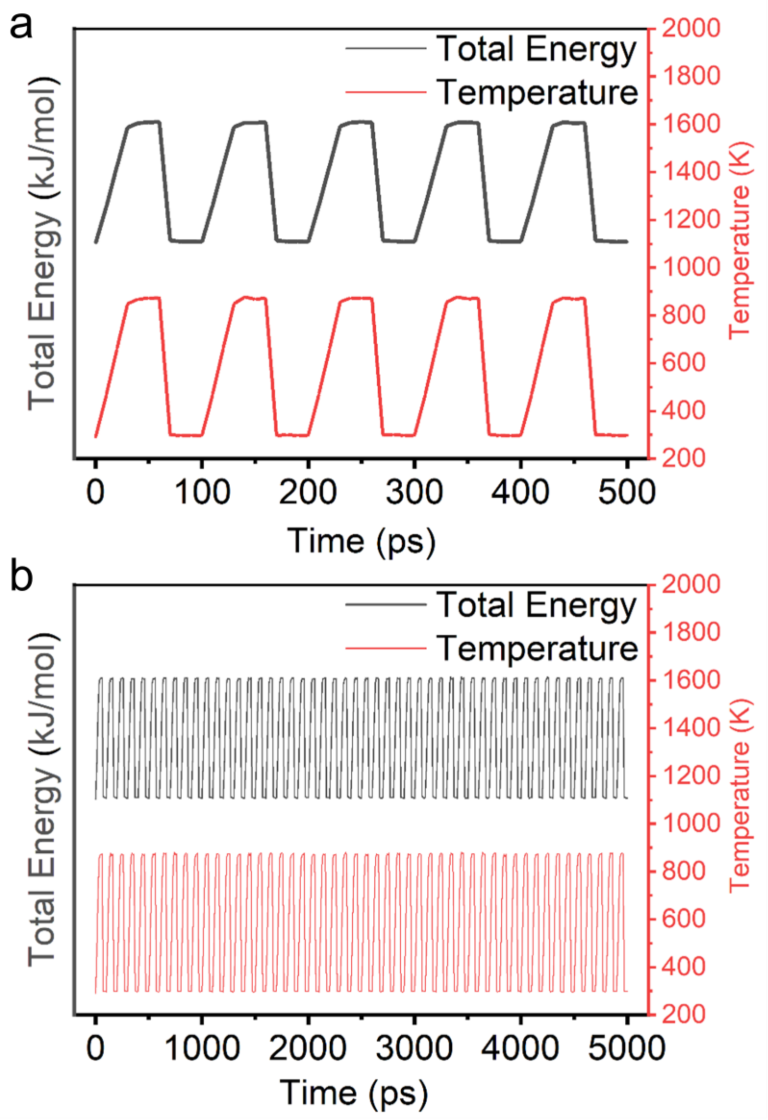

Supplementary Fig. 23. Temperature and total system energy change during heating-annealing process. (a) heating-annealing time range from 0 to 500ps; (b) heating-annealing time range from 0 to 5000 ps.

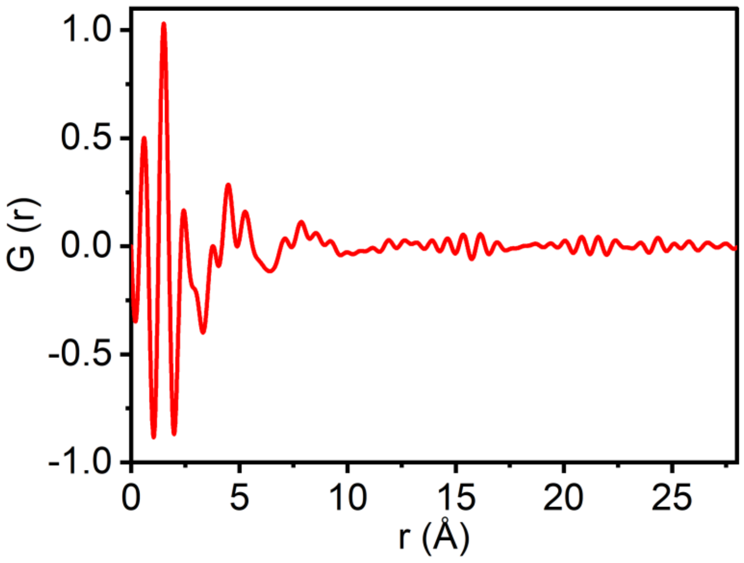

Supplementary Fig. 24. Pair distribution function of **HOF-g**.

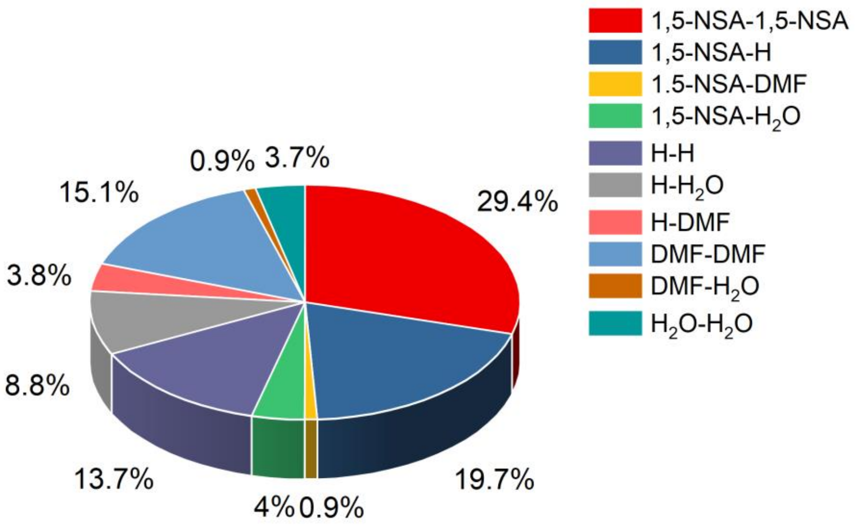

Supplementary Fig. 25. Proportion of interaction energy between different groups of components to total interaction energy.

Section 3 Supplementary References

1. W. Yang, et al. Microporous Diaminotriazine-Decorated Porphyrin-Based Hydrogen-Bonded Organic Framework: Permanent Porosity and Proton Conduction. *Cryst. Growth Des.* **16,** 5831-5835 (2016).
2. A. Karmakar, et al. Hydrogen-Bonded Organic Frameworks (HOFs): A New Class of Porous Crystalline Proton-Conducting Materials. *Angew. Chem. Int. Ed.* **55,** 10667-10671 (2016).
3. G. Xing, T. Yan, S. Das, T. Ben & S. Qiu Synthesis of Crystalline Porous Organic Salts with High Proton Conductivity. *Angew. Chem. Int. Ed.* **57,** 5345-5349 (2018).
4. Z.-B. Sun, et al. A path to improve proton conductivity: from a 3D hydrogen-bonded organic framework to a 3D copper-organic framework. *New Journal of Chemistry* **43,** 10637-10644 (2019).
5. Q. Yang, et al. Three Hydrogen-Bonded Organic Frameworks with Water-Induced Single-Crystal-to-Single-Crystal Transformation and High Proton Conductivity. *Cryst. Growth Des.* **20,** 3456-3465 (2020).
6. Y. Wang, et al. Guest-tuned proton conductivity of a porphyrinylphosphonate-based hydrogen-bonded organic framework. *J. Mater. Chem. A* **9,** 2683-2688 (2021).
7. B.-B. Hao, X.-X. Wang, C.-X. Zhang & Q. Wang Two Hydrogen-Bonded Organic Frameworks with Imidazole Encapsulation: Synthesis and Proton Conductivity. *Cryst. Growth Des.* **21,** 3908-3915 (2021).
8. S. Dekura, M. Mizuno & H. Mori Isotropic Anhydrous Superprotonic Conductivity Cooperated with Installed Imidazolium Molecular Motions in a 3D Hydrogen-Bonded Phosphate Network. *Angew. Chem. Int. Ed.* **61,** e202212872 (2022).
9. J. Yang, et al. Guest-induced proton conductivity of two-dimensional layered hydrogen-bonded organic frameworks. *Inorg. Chem. Front.* (2023).
10. X.-L. Wang, et al. Boosting Proton Conductivity in Hydrogen-Bonded Organic Layers by Modulating Embedded Guest Molecules. *Cryst. Growth Des.* **23,** 6221-6227 (2023).
11. X.-L. Wang, L. Liang, S. Dou & X.-M. Zhang Two-in-one tecton strategy to construct single crystalline hydrogen-bonded organic framework with high proton conductivity above 100 °C. *Sci. China Chem.* **66,** 2563-2568 (2023).
